# Choriocarcinoma After Full-Term Pregnancy: A Case Report and Review of the Literature

**DOI:** 10.7759/cureus.22200

**Published:** 2022-02-14

**Authors:** Emmanouil Katsanevakis, Alice Oatham, Darly Mathew

**Affiliations:** 1 Obstetrics and Gynaecology, United Lincolnshire Hospitals National Health Service Trust, Lincoln, GBR; 2 Obstetrics and Gynaecology, Chesterfield Royal Hospital, Chesterfield, GBR

**Keywords:** invasive molar pregnancy, gestational trophoblastic neoplasia, post-partum hemorrhage, gestational trophoblastic diease, choriocarcinoma

## Abstract

Choriocarcinoma is a disease associated with uncontrollable proliferation and malignant change of cells of the placenta and belongs to the malignant end of the spectrum in gestational trophoblastic disease. These tumours are usually developed after molar pregnancies, and their incidence after full-term pregnancies is extremely rare.

We present a very rare case of a 30-year-old lady, admitted with a five-month history of vaginal bleeding after a normal pregnancy. The human chorionic gonadotropin (hCG) was at a level of 209,566. A pelvic ultrasound scan revealed an endometrial thickness of 6 cm and the presence of an intra-uterine mass measuring 56 × 50 × 45 mm. After discussion with the regional gestational trophoblastic disease centre, we proceeded to a surgical evacuation of the uterus, which confirmed a post-partum choriocarcinoma (International Federation of Gynaecology and Obstetrics (FIGO) score 9). Care was continued in the specialised centre with multi-agent chemotherapy. The response was excellent, and the patient was subsequently discharged after 10 cycles of chemotherapy, and a 10-year follow-up was arranged.

Choriocarcinomas after full-term pregnancies are a rare entity. Even when they happen, they are usually associated with pregnancy complications in the ante-natal period. The prognosis is usually very good, provided that prompt diagnosis and referral to a specialised centre are made. Low-risk patients are usually treated with methotrexate monotherapy, whereas high-risk women would normally require multi-agent chemotherapy. The diagnosis of choriocarcinoma might be proven challenging even for experienced clinicians. Women should be informed that the prognosis is usually excellent, provided that they receive the right treatment.

## Introduction

Gestational trophoblastic disease (GTD) envelops a variety of diseases, from the pre-malignant conditions of complete hydatiform mole (CHM) and partial hydatiform mole (PHM), to malignant invasive mole, choriocarcinoma (CC) and more rarely, placental site trophoblastic tumour (PSTT) or epithelioid trophoblastic tumour (ETT). Collectively, the malignant forms are referred to as gestational trophoblastic neoplasia (GTN). The incidence of molar pregnancies (CHM/PHM) is reported to be 0.2-1.5 per 1000 births in Europe [1] and one in 714 in the U.K [2]. GTD in the majority of cases is related to molar pregnancies, and the incidence after a full-term pregnancy is rare. In particular, the incidence of CC after a full-term pregnancy is reported in some literature to be as rare as one in 160,000 [3].

## Case presentation

A 30-year-old G1P1 female presented to a district general hospital with a five-month history of heavy post-partum bleeding. She reported having passed large clots in the preceding few days, prompting attendance at the accident and emergency department. Her general practitioner had inserted a Mirena IUS (Bayer HealthCare Pharmaceuticals Inc., Turku, Finland) at 12 weeks post-partum, which failed to cease the bleeding. The general practitioner then proceeded to prescribe norethisterone pills one month prior to admission.

The index pregnancy was normal, with no associated complications. A healthy baby was born vaginally with a ventouse and was being exclusively breastfed. There had been no reported nausea, vomiting, cough, abdominal or pelvic pain, nor loss of consciousness associated with the erratic and heavy bleeding. The patient had only partaken in sexual intercourse once in the post-partum period, with the Mirena IUS in-situ. There was no notable family history. The patient had a previous appendectomy at age 19 and a borderline smear cytology result four years previously with no treatment.

The general examination was largely unremarkable, with no abdominal tenderness; however, the uterus was palpable at 12 weeks' size. Normal vulva and vagina, with some large clots and blood noted in the vaginal canal. The cervical os was closed. There were no focal neurological signs nor other signs of any potential metastatic disease. One gram of tranexamic acid was administered, which slowed the bleeding. Blood results demonstrated haemoglobin of 121 and a beta-human chorionic gonadotropin (b-hCG) of 209,566.

**Figure 1:**
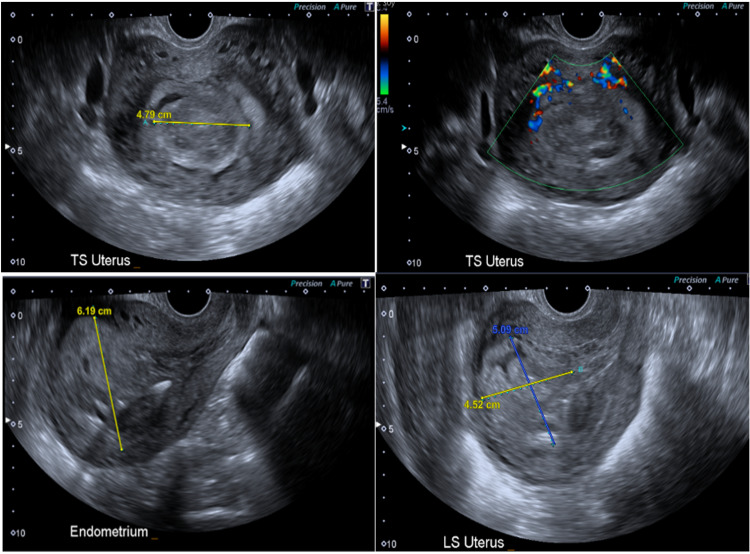
Sonographic findings. TS: transverse section; LS: longitudinal section.

A trans-vaginal ultrasound scan demonstrated a thickened endometrium measuring 62 mm and a large mixed echoic mass in the cavity measuring 56 × 50 × 45 mm with some vascularity (Figure 1). Mirena IUS in-situ, with the rest of the findings being non-remarkable. Given the preliminary diagnosis of gestational trophoblastic disease, a chest x-ray (CXR) was performed to rule out any early pulmonary metastases; this was clear. An MRI scan of the head was also performed, and that showed normal intracranial appearances and no visible metastases.

Her International Federation of Gynaecology and Obstetrics (FIGO) score was 9 (high risk): <40 years old (30 = 0), index pregnancy (normal = 2), time since delivery (five months = 1), b-hCG (209,566 = 4), size of tumour (>50 mm, retained products of conception (RPOC) = 2), metastases (none on CXR = 0).

The case was discussed with the local GTD centre and it was agreed that the most likely differential was that of post-partum choriocarcinoma. The patient then proceeded to a surgical evacuation of the uterus on the advice of the local GTD centre. Products of conception (POC) were obtained and sent for histological analysis.

Peri-operatively, the patient lost an estimated 1.5 l of blood and received two units of packed red cells. The POC were felt to be friable on palpation and likely molars.

Histology demonstrated macroscopically a sac weighing 40.6 g, with no obvious vesicles nor fetal parts. Microscopically, it was comprised of large areas of haemorrhage with some necrosis and viable cells. The viable cells were in the form of atypical mononuclear intermediate trophoblastic cells in sheets that were surrounded by multinucleate syncytiotrophoblast tumour cells. There was no myometrial invasion, nor were chorionic villi visualised. The pattern was consistent with a diagnosis of choriocarcinoma. All cells stained strongly positive for programmed death ligand 1 (PDL1). There were some tumour-infiltrating lymphocytes (CD3 and CD8 positive).

Following discharge, the patient presented with a further episode of blood loss; approximately a 120-ml sized clot.

**Figure 2:**
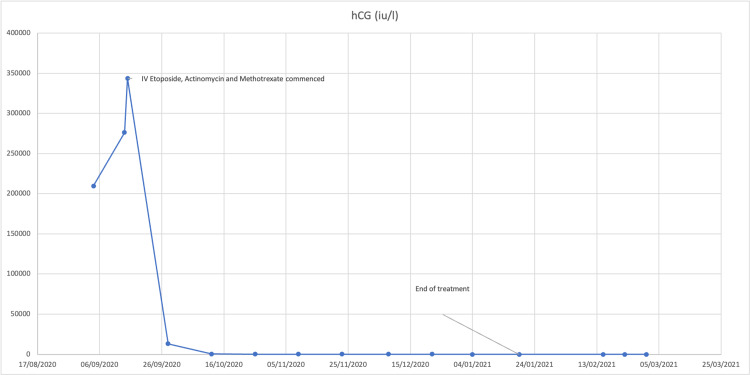
hCG trend over time. hCG: human chorionic gonadotropin.

The patient was transferred to the local GTD centre with the diagnosis of post-partum choriocarcinoma and she received 10 cycles of chemotherapy (etoposide, methotrexate (MTX), and actinomycin). She responded very well to chemotherapy and was advised that she could try to conceive again after one year. She will be followed-up in the tertiary centre for 10 years. Her b-hCG results are demonstrated in Figure 2.

## Discussion

Choriocarcinoma after term pregnancy is a rare entity [4], with an estimated incidence after a live birth of one in 50,000 [5,6]. Diagnosis is usually prompted by increased levels of hCG in association with post-partum haemorrhage, like in our case. Final confirmation of the diagnosis is established after histological examination of the placenta or endometrial currettings. However, in many cases, the diagnosis is incidentally established after histopathological examination of the placenta [7]. Placentas are usually macroscopically normal and a diagnosis is reached after microscopic examination. There are also reported cases of choriocarcinoma in macroscopically abnormal placentas containing small lesions or nodules that suggest infarcts.

Choriocarcinomas, unlike our case, tend to be associated with pregnancy complications such as hydrops fetalis, fetal-maternal haemorrhage, intra-uterine fatal death, and irregular cardiotocography (CTG) patterns. In our case, the pregnancy was uncomplicated. Maternal complications may include intracranial haemorrhage and pulmonary embolism, thus choriocarcinoma should be considered as a possible diagnosis in women who present with these pathologies, especially in the post-natal period [4].

The prognosis of choriocarcinoma is usually very good, provided that the appropriate referral to a tertiary centre is made and the appropriate chemotherapy regimen is started in a timely manner [8,9]. However, the prognosis might be worse in cases of CC after non-molar pregnancy, probably due to delay in diagnosis or advanced disease [10]. The FIGO prognostic score is widely used to identify high-risk patients and guide treatment regimens [2]. Low-risk patients (FIGO 6 or less) are usually treated with single-agent chemotherapy (methotrexate), whereas high-risk patients (FIGO 7 or more) require polytherapy with etoposide, methotrexate, and dactinomycin being one of the most widely used treatment regimens. The cure rate after successful referral to a tertiary centre is 98-100% in the U.K [2]. High dose chemotherapy with stem cell recovery might be required in rare cases of multi-relapsed disease [11]. Confirming the diagnosis with tissue diagnosis is a matter of debate and discussion, as the diagnosis is often evident from the history, the clinical picture, and the hCG levels. However, it can sometimes lead to different treatments, in case the placental site trophoblastic tumour is diagnosed. Proceeding to a surgical evacuation of the uterus to confirm the diagnosis of choriocarcinoma is also associated with an increased risk of bleeding [2], as in our case, thus effective communication with the local gestational trophoblastic centre should be maintained.

In regard to the long-term outcomes, these are generally promising, with approximately 80% of women achieving a further pregnancy following MTX treatment or multi-agent chemotherapy [2]. Women, on the other hand, should be warned about the risk of early menopause and the implications for fertility as they approach the age of 40 [12]. Finally, the potential risk of a second cancer is extremely low. One large study [12] has reported no increase in the risk of cancer after MTX or multi-agent chemotherapy treatment.

## Conclusions

In summary, diagnosing CC can be challenging. However, combining the gynaecological history, elevated b-hCG levels and USS findings, usually leads to the diagnosis. Consideration should be given, as to whether or not a tissue biopsy is needed before starting treatment. Biopsy often leads to heavy bleeding, like in our case, and if the diagnosis is evident, it could be avoided. The overall prognosis is very good if a prompt diagnosis is made and care is provided in a centre with experience in the management of these cases.
